# Rules of engagement: Reactions to internal and external criticism in public debate

**DOI:** 10.1111/bjso.12351

**Published:** 2019-11-06

**Authors:** Levi Adelman, Maykel Verkuyten

**Affiliations:** ^1^ Utrecht University The Netherlands

**Keywords:** group criticism, intergroup sensitivity effect, refugees, social identity

## Abstract

Since 2014, the refugee crisis has launched a political shockwave across Europe, with consequences for the European Union, the Schengen Zone, and national politics. Within this context, we investigated how public statements about the refugee crisis are received. While debate and criticism are hallmarks of a democratic society, research demonstrates that people respond more negatively to criticism about their group from an outsider compared with an insider. But does this reflect a protective bias in favour of one’s own group, or a more principled position against criticism from outsiders independently of one’s own group membership? In three experimental studies, people apply the principle of preferring internal over external criticism, even to the point of penalizing in‐group members who criticized outgroups. This preference for internal over external criticism is guided by perceptions that internal critics are more constructive and more expert than external critics.

## Background

The United Nations High Commissioner for Refugees estimates that just under two million refugees have entered Europe through the Mediterranean since 2014 (UNHCR, 2019). During that time, Europe has seen political upheavals over how to respond to this influx with strong debates in all societies, including the Netherlands where the current research was conducted (Harteveld, Schaper, De Lange, & Van Der Brug, 2018). In this polarized debate, some people emphasize the need to support these newcomers and criticize the lack of hospitality and humanitarianism, while others favour border closure and claim that the newcomers are ‘fortune seekers’ who endanger the stability of society (Van Prooijen, Krouwel, & Emmer, 2018; Verkuyten, Mepham, & Kros, 2018). Research has found that openness to critical messages about one’s own group may turn not on the content of the message but on the identity of the critic (Hornsey, Oppes, & Svensson, 2002; Jetten & Hornsey, 2014), with an external group critic being perceived more negatively and their message being more rejected. But it is unclear whether this reflects preferential treatment for in‐group members who are permitted to criticize compared with outsiders, or whether it reflects a social norm that criticism should come from insiders and not from outsiders, independent of one’s own group membership (Sutton, Elder, & Douglas, 2006).

Within the framework of the so‐called ‘refugee crisis’ debate, we examined these two accounts for the preferential treatment of internal versus external critics in three studies using ethnic Dutch national samples. Critical for testing the contrasting predictions, we used a between‐subjects survey‐embedded experimental design in which all combinations of intergroup (external) and intragroup (internal) criticism were presented to participants. We also examined perceived constructiveness of the criticism and perceived expertise of the critic as underlying mechanisms (Studies 2 and 3).

### Political discourse as a social identity process

Research suggests that despite the importance of openness to critical and dissenting opinions, people appear to use group‐identity‐based judgements in determining their response to in‐group criticism. Hornsey *et al. *(2002) tested people’s reactions to both praise and criticism of their group coming from either an in‐group or an outgroup source. They found that while people are equally open to positive messages about their group regardless of the source, they respond more defensively to critical messages from an outgroup rather than an in‐group critic (see also Elder, Sutton, & Douglas, 2005; also Jetten & Hornsey, 2014 for a review).

By one interpretation, this intergroup sensitivity effect (ISE) emerges from social identity processes (Tajfel & Turner, 1979) whereby people are motivated to value and protect their in‐group. There is a large literature demonstrating that people give preferential treatment to in‐group members in terms of evaluations (Ratner, Dotsch, Wigboldus, van Knippenberg, & Amodio, 2014), attributions (Hewstone, 1990), and behaviour (Greenwald & Pettigrew, 2014). Whereas praise contributes to a positive in‐group image and is therefore equally well‐received from in‐group and outgroup members (Rabinovich, Morton, Crook, & Travers, 2012), criticism can harm the positive in‐group image and is generally less well‐received, but especially when coming from outsiders who challenge the meaning and value of one’s in‐group.

Compared with outgroup critics, in‐group critics are perceived as delivering more constructive criticism and as being more expert in issues related to the in‐group (Hornsey & Imani, 2004), and particularly constructiveness underlies the intergroup sensitivity effect (Hornsey & Imani, 2004; Sutton, Elder, & Douglas, 2006). These judgements about constructiveness and expertise appear to be made on the basis of group identification. For example, a perceived lack of experience with the in‐group can limit openness to an in‐group critic (Hornsey & Imani, 2004), and if the in‐group critic is described as having a weak connection to the group, the preferential treatment of that in‐group critic disappears (Hornsey, Trembath, & Gunthorpe, 2004). Thus, the literature finds that although people generally dislike hearing criticism about their own group, they prefer it from fellow in‐group members, and this is primarily because they are considered more constructive in their motives than outgroup members. However, it is not clear whether this preference reflects social identity concerns whereby people are inclined to protect the in‐group from negative judgements by outsiders, or rather a more normative position about group criticism (‘one should only criticize one’s own group’) that is applied to all critics independently of one’s own group membership, and would be reflected in negative responses even to in‐group members who criticize other groups. This is particularly critical in the context of the ongoing refugee crisis, where public opinion is divided between seeing migrants as refugees who deserve support or as illegal immigrants who are threatening the security of host societies, and thus includes denunciations both against host societies and refugee populations.

### Norm of preferring internal over external criticism

To investigate the possibility that the preference for internal critics reflects a norm that internal but not external criticism is acceptable, Sutton *et al. *(2006) tested how third‐party observers (British participants) would respond to Australians being criticized by fellow Australians or by Americans, Canadians, or New Zealanders. As uninvolved observers, the British participants would not be expected to display any in‐group favouritism. Across two studies, they found that the participants displayed a preference for internal over external critics regardless of group membership which suggests that they applied a more broadly applicable norm. Sutton *et al. *(2006) argue that this norm reflects people’s expectation that, in general, internal criticism can reflect caring about one’s group, while external criticism is an attack on the target outgroup. In a third study, they also found that people broadly agreed with global statements that internal criticism is more acceptable than external criticism.

However, the existing research leaves open the possibility that while people may generally agree with the norm that criticism should be internal and not external, people may not necessarily practice this rule when it affects them personally. Another study sought to address this issue (Sutton, Douglas, Elder, & Tarrant, 2008) by having both in‐group and outgroup critics and criticism of both the in‐group and the outgroup. Results, however, were mixed and could not determine whether people prefer to protect their in‐group from external criticism while not affording outgroups the same protection. However, Lindner and Nosek (2009, Study 2) found evidence in one study that, independent of implicit ethnic in‐group preferences, people were more willing to protect a critical statement when the speaker was a member of the criticized group (White American criticizing Americans and Arab American criticizing Arabs).

Thus, two competing perspectives on the intergroup sensitivity effect suggest different predictions about how people will respond to in‐group and outgroup critics. The social identity perspective, which argues that being an in‐group member tends to generate intergroup biases because in‐group members are trusted more and therefore tend to be evaluated more positively compared with outgroup members (but see the research into the black sheep effect, Marques, Yzerbyt, & Leyens, 1988, and dissent from the group, Packer, 2009), would seem to predict (Hypothesis 1) that the most positive reaction exists in the setting in which an in‐group member criticizes their own either in‐group or the outgroup, compared with an outgroup member criticizing their in‐group or the outgroup (in‐group critic > outgroup critic).

Alternatively, the norm against external criticism leads to the prediction (Hypothesis 2) that criticism within an intragroup context will be evaluated more positively than within an intergroup context (internal critic > external critic). Importantly, internal critics should be evaluated equally positively regardless of the group membership of the responding individual. In other words, individuals will be more accepting of a critic in an intragroup versus intergroup context, independently of the respondents’ own group membership.

The present research seeks to test these two predictions by examining ethnic Dutch participants’ evaluations of criticism directed at either the in‐group (the Dutch) or the outgroup (refugees) by either an in‐group (ethnic Dutch‐named) or outgroup (Middle Eastern‐named) critic. While much of the past research into the ISE has looked at group criticisms separated from politically heated topics, we focus on the context of the refugee crisis in Europe that mainly involves people fleeing Middle Eastern countries (Syria, Iraq). By making the critical messages about assigning blame for the controversial reception of refugees in the Netherlands to either the Dutch or to refugees, the ethnic Dutch participants are put into a potentially difficult position. If they are acting in accordance with the norm of internal rather than external criticism, they should not only reject external criticism targeting their own group but also reject in‐group members’ criticism of refugees. But if participants make their evaluations based on social identity concerns, they would favour ethnic Dutch speakers both when delivering critical messages about the in‐group and about the outgroup (refugees), whereas the outgroup critic should be perceived generally less favourably.

## STUDY 1

In Study 1, we used an experimental design in with all combinations of in‐group and outgroup criticisms and critics were systematically varied. As research suggests that contexts such as audience size and composition can affect the intergroup sensitivity effect (Elder, Sutton, & Douglas, 2005), we also tested whether expressing the criticism on one’s private property or in a public community centre influences the acceptability of the message. We expected that people would be more favourable to private rather than public criticism, and we explored whether this would depend on the identities of the critic and the target.

## Method

### Participants

A representative sample of 815 self‐identified ethnic Dutch participants was collected as part of an Internet survey about culture and identity.1Other topics covered in the survey included intergroup tolerance, intergroup attitudes, and national nostalgia. Six participants were excluded for not being born in the Netherlands, and one Muslim‐identifying participant was also excluded. The remaining 808 participants were 52.5% male and 47.5% female, with an average age of 52.9 (*SD* = 16.64), ranging from 18 to 92 years old, and on a political self‐placement scale (Jost, 2006) tended to lean towards the centre‐right with 12.8% self‐identifying as left‐leaning, 16.1% as centre‐left, 24.9% as centre, 21.2% as centre‐right, and 12.0% as right. An additional 13.1% declined to state a political orientation.

### Design and measures

We used a 2 (critic ethnicity) × 2 (target of criticism) × 2 (location of message) between‐subjects random design. Following Lindner and Nosek (2009), participants read a brief description of an act of criticism, modelled on a newspaper‐style article about citizens’ responses to the challenges and problems that the reception of refugees poses and in which either the Dutch or refugees are considered ‘the problem’. The scenario described a situation in which someone in the city of Gouda in the Netherlands had put up a poster and there were three independent variables that differed between the stories. First, the person putting up the poster was always described as having been born, raised, and employed in the Netherlands, but was either given a typical Dutch male name (Johan Kok) or a Middle Eastern name (Yusuf Hamdi2This name was selected as representing a common Middle Eastern first and last name. This combination is not associated with any specific known individuals.), which we expected to be linked to (mainly Middle Eastern) refugees by our Dutch participants. We focused on males because males, and especially Middle Eastern males, are much more likely to be involved in politics than females. Second, the poster content either read that ‘The Dutch are the problem’ or ‘Refugees are the problem’. Third, the poster had either been hung in the front window of a house (private space) or the window of a community centre (public space).

#### Outcome variables

Response scales ranged from 1 (*Totally Disagree*) to 7 (*Totally Agree*). First, as the main purpose of a communication is persuasion, participants indicated their agreement or disagreement with the message using a single item, ‘I agree with his opinion’ (*M* = 3.21, *SD* = 1.54). Next, to measure how acceptable the message was, participants indicated using two items whether they thought expressing such an opinion and engaging in such actions should be allowed (*r* = .70, *M* = 4.20, *SD* = 1.38): ‘It must be allowed for him to give his opinion in this way’ and ‘The local residents must accept his action’. Two items also measured participants’ willingness to interfere and have the message removed (*r* = .71, *M* = 4.67, *SD* = 1.34): ‘He should remove the poster to prevent neighbours from feeling offended and hurt’ and ‘He should remove the poster to prevent tensions and conflicts in the neighbourhood’.3Factor analyses on the DVs revealed inconsistent results, suggesting a single factor in Study 1, and different two‐factor solutions in Studies 2 and 3. Therefore, we present the results in the proposed theoretical constructs. Finally, ethnic Dutch identification was measured with a single item (Postmes, Haslam, & Jans, 2013), ‘How strongly do you feel Dutch’, on a 1–10 scale (*M* = 8.53, *SD* = 1.65).

## Results

All three independent variables were entered into an omnibus ANOVA and all unreported main effects and interactions were non‐significant, including all the effects for the location of the poster. Ethnic Dutch identification and political self‐placement did not meaningfully affect the results below.4When entered as a full factorial variable, there were no effects of ethnic Dutch identification. However, political self‐placement interacted with the identity of the critic, with conservatives (+1 *SD*) responding more positively to Dutch than Middle Easterner critics and liberals (−1 *SD*) not differentiating between them, and the target of the criticism, with liberals preferring when the Dutch are blamed, and conservatives preferring when refugees are blamed.


### Opinion agreement

**Figure 1:**
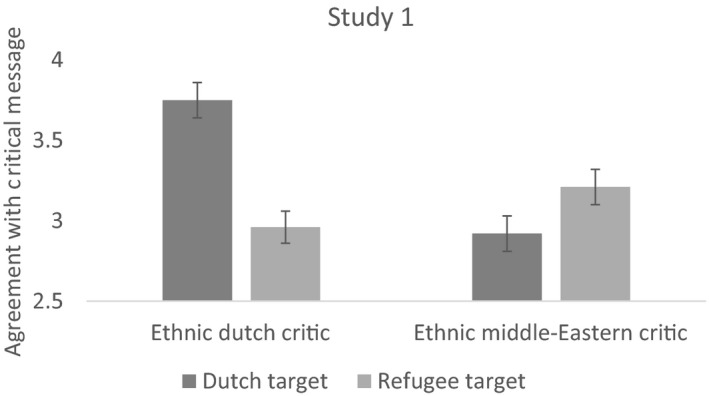
The pattern of findings for intragroup criticism (an ethnic Dutch criticizing the Dutch or a Middle Easterner criticizing refugees) and intergroup criticism (an ethnic Dutch criticizing refugees or a Middle Easterner criticizing the Dutch). Error bars represent standard errors (*SE*), and the outcome variable (*y*‐axis) was measured on a 1–7 scale.

Main effects of critic ethnicity, *F*(1, 800) = 7.24, *p* = .007, $\eta_{p}^{2}$ = .009, and message target, *F*(1, 800) = 5.30, *p* = .022, $\eta_{p}^{2}$ = .007, show that participants agree more with an ethnic Dutch (*M* = 3.35, *SD* = 1.61) rather than Middle Eastern (*M* = 3.07, *SD* = 1.47) critic and agree more when the target is Dutch people (*M* = 3.32, *SD* = 1.47) rather than refugees (*M* = 3.09, *SD* = 1.61). These effects were qualified by an interaction between critic and target group, *F*(1, 800) = 25.45, *p* < .001, $\eta_{p}^{2}$ = .031 (see Figure 1), such that people agreed more with a Dutch critic who criticized Dutch people (*M* = 3.75, *SE* = .11) rather than refugees (*M* = 2.96, *SE* = .11), *t*(800) = −5.17, *p* < .001, *d* = −.37, and they also agreed marginally more with a Middle Eastern critic who criticized refugees (*M* = 3.21, *SE* = .11) rather than the Dutch (*M* = 2.92, *SE* = .10), *t*(800) = 1.95, *p* = .051, *d* = .14. Thus, the pattern indicates greater agreement with intragroup rather than intergroup criticism, supporting the norm of internal over external criticism.

### Permitting the message

Main effects of critic ethnicity, *F*(1, 800) = 15.68, *p* < .001, $\eta_{p}^{2}$ = .019, and of the message target, *F*(1, 800) = 4.94, *p* = .027, $\eta_{p}^{2}$ = .006, show that people are more permissive of a Dutch (*M* = 4.39, *SD* = 1.37) rather than Middle Eastern (*M* = 4.01, *SD* = 1.36) critic, and when the target is Dutch people (*M* = 4.29, *SD* = 1.39) rather than refugees (*M* = 4.10, *SD* = 1.36). However, an interaction between ethnicity of the critic and the target of the criticism, *F*(1, 800) = 27.73, *p* < .001, $\eta_{p}^{2}$ = .034, showed that people were more permissive towards a Dutch critic who criticized Dutch people (*M* = 4.75, *SE* = .10) rather than refugees (*M* = 4.04, *SE* = .09), *t*(800) = −5.26, *p* < .001, *d* = −.37, and they were more permissive towards an Middle Easterner criticizing refugees (*M* = 4.16, *SE* = .10) rather than the Dutch (*M* = 3.88, *SE* = .09), *t*(800) = 2.16, *p* = .031, *d* = .15.

### Restricting the message

Finally, a main effect of critic ethnicity, *F*(1, 800) = 8.23, *p* = .004, $\eta_{p}^{2}$ = .010, found that participants were more likely to want the poster taken down when the critic was an ethnic Middle Easterner (*M* = 4.80, *SD* = 1.33) than ethnic Dutch (*M* = 4.53, *SD* = 1.34). However, a significant interaction between critic and target, *F*(1, 800) = 13.35, *p* < .001, $\eta_{p}^{2}$ = .016, indicated that participants were less in favour of restricting the message of an ethnic Dutch critic when it targeted fellow Dutch people (*M* = 4.35, *SE* = .09) rather than refugees (*M* = 4.71, *SE* = .09), *t*(800) = −2.70, *p* = .007, *d* = .19, whereas participants were less in favour of restricting the message of a Middle Eastern critic when it targeted refugees (*M* = 4.64, *SE* = .09) rather than Dutch people (*M* = 4.96, *SE* = .09), *t*(800) = −2.46 *p* = .014, *d* = −.17.

### Internal vs. external criticism

Across the outcome variables, findings indicate that an ethnic Dutch critic was better received when criticizing the Dutch rather than refugees, while a Middle Eastern critic was better received when criticizing refugees rather than the Dutch. In line with Hypothesis 2, this suggests that internal versus external criticism, rather than the identity of the critic relative to the respondents, determines people’s responses. Therefore, to further focus on this difference between internal and external criticism, we created a new independent variable with two conditions representing those who criticized the in‐group (ethnic Dutch who blamed the Dutch and Middle Easterner who blamed refugees) and those who criticized the outgroup (ethnic Dutch who blamed refugees and Middle Easterner who blamed the Dutch) and focused on how participants respond to internal versus external criticism.

As expected, people agree more with internal (*M* = 3.48, *SD* = 1.51) than external critics (*M* = 2.94, *SD* = 1.54), *t*(806) = 5.05, *p* < .001, *d* = .36. The critical messages also generate greater permission from internal (*M* = 4.45, *SD* = 1.31) rather than external (*M* = 3.95, *SD* = 1.40) critics, *t*(806) = 5.28, *p* < .001, *d* = .37, and internal critics generate less restriction (*M* = 4.49, *SD* = 1.32) than external critics (*M* = 4.84, *SD* = 1.33), *t*(806) = −3.72, *p* < .001, *d* = −.26.5To further test whether this was a general principle, we tested whether the internal–external difference was moderated or otherwise affected by gender. Across all three studies, gender did not significantly moderate these effects consistently, offering further support for a general principle. See the Supporting Information for more details.


## Discussion

The findings of Study 1 indicate that in the context of the refugee crisis, internal criticism is more accepted than external criticism. The pattern of findings supports the hypothesis that people show preference for internal critics and therefore seem to act upon a normative position which prefers internal over external criticism rather than displaying in‐group favouritism.

## STUDY 2

The aim of Study 2 was to replicate this pattern of findings and to examine two key mechanisms that have been proposed to underlie the intergroup sensitivity effect. First, critical messages from in‐group members are perceived to be more constructive than those from outgroup members, because in‐group members are expected to have more positive motives and intentions towards their in‐group (Adelman & Dasgupta, 2019; Hornsey & Imani, 2004). Second, in‐group compared with outgroup members are perceived to be more likely to base their criticism on in‐group knowledge and experience. A person from the in‐group would therefore be judged as better informed than outgroup members, leading people to trust their commentary and judgement more (Hornsey & Imani, 2004; Wilson & Sherrell, 1993).

Following the norm of preferring in‐group over outgroup criticism, we would expect that internal critics (ethnic Dutch criticizing the Dutch and Middle Easterners criticizing refugees) are considered better informed and more constructive than external critics (ethnic Dutch criticizing refugees and Middle Easterner criticizing the Dutch). By contrast, from a social identity perspective we would expect that critics from one’s own group (Dutch) would be seen as more constructive and better informed than critics from the outgroup (Middle Eastern), and especially by higher ethnic Dutch identifiers. Additionally, we also added a manipulation check to Study 2 in which we asked respondents about the critics’ feelings towards refugees, to check whether participants were recognizing and understanding the information in the manipulations.

## Method

### Participants

A total of 450 responses were collected in a large, representative Internet survey of ethnic Dutch participants on immigration and intergroup attitudes.6Other topics covered in the survey included Brexit attitudes, social tolerance beliefs, and the slippery slope fallacy. Forty‐six were excluded for not having both parents born in the Netherlands, leaving 404 non‐Muslim ethnic Dutch participants for analysis. Participants were 51.0% male and 49.0% female, with an average age of 51.7 years (*SD* = 16.9) with a range of 18–85 years, and politically tended to lean towards the centre‐left with 15.1% self‐identifying as left‐leaning, 16.8% as centre‐left, 30.2% as centre, 14.1% as centre‐right, and 9.7% as right. An additional 14.1% declined to respond.

### Measures

The manipulation in Study 2 was identical to that of Study 1, except that we did not manipulate the location where the poster was placed. Thus, a 2 (critic ethnicity: ethnic Dutch vs. Middle Easterner) × 2 (target of blame: Dutch people vs. refugees) design was used.

The outcome and ethnic Dutch identification measures in Study 2 were identical to those in Study 1: agreement with the stated opinion (*M* = 3.24, *SD* = 1.58), permitting the message (*r* = .66, *M* = 4.59, *SD* = 1.38), restricting the message (*r* = .73, *M* = 4.56, *SD* = 1.41), and ethnic Dutch identification (*M* = 8.49, *SD* = 1.52). Before the outcome measures, participants were asked to indicate (7‐point scales; partially adapted from Hornsey & Imani, 2004) the extent to which they believed that the critic was making a constructive contribution to the debate over refugees7Note that this single‐item measure of constructiveness differs from the way that constructiveness is assessed in previous work (Adelman & Dasgupta, 2019; Hornsey & Imani, 2004). In previous work, the measure of constructiveness asked explicitly about the mindset and intentions of the critic, whereas here the measure is about the perceived constructiveness of the message. (‘To what extent do you think that [critic]'s action makes a positive contribution to the debate on refugees?’; *M* = 3.05, *SD* = 1.60) and whether the critic had a well‐informed position (‘To what extent do you think [critic] is well informed about the refugee issue?’; *M* = 3.50, *SD* = 1.43).

### Manipulation check

As a manipulation check, participants were asked to indicate the critic’s feelings about refugees (‘What do you think are [critic]’s feelings towards refugees?’; *M* = 3.66, *SD* = 1.91). The aim of this measure was to test whether the joint manipulations of critic and target identity were understood as intended. Therefore, we measured the construct of perceived liking which is distinct from the outcome measures of one’s own agreement or permission that motivated this research. Based on the intergroup criticism literature (Hornsey, Oppes, & Svensson, 2002), the manipulation of the criticism should mean that those who blame refugees are perceived to have more negative attitudes and that people perceive external critics (Dutch) to hold more negative attitudes overall. More importantly, these two effects should intersect, such that those blaming refugees are thought to hold more negative attitudes towards them when they are external rather than internal critics, with little to no difference when blaming the other party (the Dutch). We indeed found that critics who blamed refugees (*M* = 2.47, *SD* = 1.37) were considered to have less positive feelings about refugees than those who blamed the Dutch (*M* = 4.99, *SD* = 1.52), *F*(1, 400) = 325.85, *p* < .001, $\eta_{p}^{2}$ = .449. Additionally, the Middle Eastern critic (*M* = 3.83, *SD* = 1.84) was judged to have a more positive attitude towards refugees than the Dutch critic (*M* = 3.47, *SD* = 1.98), *F*(1, 400) = 8.03, *p* = .005, $\eta_{p}^{2}$ = .020. Furthermore, an interaction, *F*(1, 400) = 8.45, *p* = .004, $\eta_{p}^{2}$ = .021, indicated that while those who blamed the Dutch were considered to have equally positive feelings towards refugees (*M*_Dutch_ = 5.00, *SE* = .15; *M*_Middle Eastern_ = 4.99, *SE* = .14), *t*(400) = 0.05, *p* = .960, *d* < .001, the Dutch critic who blamed refugees was considered to have more negative feelings towards refugees (*M* = 2.04, *SE* = .14) than the Middle Eastern critic who blamed refugees (*M* = 2.85, *SE* = .13), *t*(400) = −4.18, *p* < .001, *d* = −.42. These results indicate that the manipulations of criticism valence and critic identity were successful.

## Results

**Figure 2:**
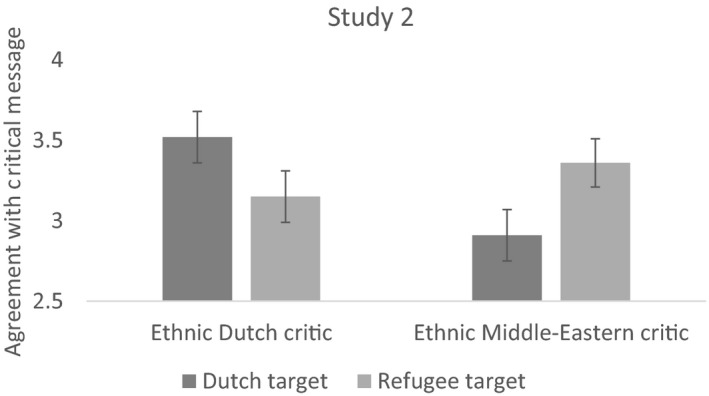
The pattern of findings for intragroup criticism (an ethnic Dutch criticizing the Dutch or a Middle Easterner criticizing refugees) and intergroup criticism (an ethnic Dutch criticizing refugees or a Middle Easterner criticizing the Dutch). Error bars represent standard errors (*SE*), and the outcome variable (*y*‐axis) was measured on a 1–7 scale.

The results were again unaffected by ethnic Dutch identification or political self‐placement.8As in Study 1, political self‐placement interacted with the identity of the target, such that liberals preferred when the Dutch are blamed while conservatives preferred when refugees are blamed. Tests of the main and two‐way interaction effects provided similar but somewhat weaker effects compared with Study 1. Specifically, the findings indicate that participants agree more with internal criticism than with external criticism, but the differences were significant for the Middle Eastern critic and not for the ethnic Dutch critic. However, the directions of the effects again suggest that people are operating, at least partly, using a norm of preferring internal over external criticism (see Figure 2 below and the Appendix S1). Following the analytic approach used in Study 1, we again created an independent variable with two conditions representing internal criticism and external criticism.

We found that internal critics generate more agreement (*M* = 3.43, *SD* = 1.55) than external critics (*M* = 3.03, *SD* = 1.59), *t*(402) = 2.57, *p* = .010, *d* = .26, and that the opinions of the internal critics also generate greater permission (*M* = 4.77, *SD* = 1.27) than external critics (*M* = 4.41, *SD* = 1.48), *t*(402) = 2.60, *p* = .010, *d* = .26, and less restriction (*M* = 4.41, *SD* = 1.33) than external critics (*M* = 4.72, *SD* = 1.48), *t*(402) = −2.24, *p* = .026, *d* = −.22.

### Testing the proposed mechanisms

An interaction between critic ethnicity and target group on constructiveness, *F*(1, 400) = 14.04, *p* < .001, $\eta_{p}^{2}$ = .034, showed that when the message came from an ethnic Dutch critic, it was perceived to be more constructive when it targeted the Dutch (*M* = 3.44, *SE* = .16) rather than refugees (*M* = 2.82, *SE* = .16), *t*(400) = 2.73, *p* = .007, *d* = .27. In contrast, when the message came from a Middle Eastern critic, it was perceived as more constructive when it targeted refugees (*M* = 3.24, *SE* = .15) rather than the Dutch (*M* = 2.68, *SE* = .16), *t*(400) = 2.57, *p* = .011, *d* = .26.

Similarly, an interaction on expertise, *F*(1, 400) = 8.29, *p* = .004, $\eta_{p}^{2}$ = .020, showed that ethnic Dutch critics were considered better informed when they blamed Dutch people (*M* = 3.78, *SE* = .15) rather than refugees (*M* = 3.17, *SE* = .14), *t*(400) = 2.98, *p* = .003, *d* = .30, although Middle Eastern critics did not differ (*M*_Dutch Target_ = 3.42, *SE* = .14; *M*_Refugee Target_ = 3.62, *SE* = .13), *t*(400) = −1.05, *p* = .294, *d* = −.11. Thus, the findings demonstrate an intragroup versus intergroup distinction whereby internal critics are considered more constructive and better informed than external critics.

Therefore, we tested the intragroup versus intergroup difference and we found that internal criticism was considered more constructive (*M* = 3.33, *SD* = 1.58) than external criticism (*M* = 2.75, *SD* = 1.57), *t*(402) = 3.69, *p* < .001, *d* = .37, and internal critics were considered to have greater expertise (*M* = 3.69, *SD* = 1.39) than external critics (*M* = 3.29, *SD* = 1.44), *t*(402) = 2.85, *p* = .005, *d* = .28.

To test the mechanism, we then entered constructiveness and expertise simultaneously into a mediation model where external rather than internal criticism predicted agreement, permission to offer the criticism, and support for restricting the criticism. Following recent recommendations (Yzerbyt, Muller, Batailler, & Judd, 2018), we conducted mediation analyses by looking for joint significance of the paths between the predictor and the mediators and the mediators and the outcome variable, and by utilizing the Monte Carlo sampling method to generate magnitudes and confidence intervals of the indirect effects. As results were similar across the three outcome variables, for reasons of brevity we only present the results for agreement here (see Appendix S1).

### Agreement with the critical message

**Figure 3:**
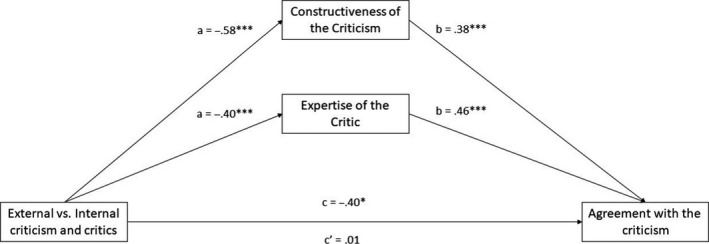
The total path of external versus internal criticism on agreement with the criticism (c path), the indirect paths leading to the two mediators (a paths), and then to the outcome variables (b paths), and the direct path from external (compared with internal) criticism on the outcome controlling for the mediators (c′ path). *p < .05, **p < .01, ***p < .001.

The total effect of external (compared with internal) criticism indicated decreased agreement with the critical message, *B* = −.40, *SE* = .16, 95% CI [−0.709, −0.095]. However, this effect was mediated through perceived message constructiveness and critic expertise, as the external (compared with internal) criticism was seen as less constructive, *B* = −.58, *SE* = .16, 95% CI [−0.884, −0.269], and as coming from a less expert critic, *B* = −.40, *SE* = .15, 95% CI [−0.678, −0.125]. In turn, both constructiveness, *B* = .38, *SE* = .04, 95% CI [0.298, 0.468], and expertise, *B* = .46, *SE* = .05, 95% CI [0.370, 0.558], predicted greater agreement with the critical message. Monte Carlo simulations revealed significant indirect effects of perceived constructiveness, *B* = −.22, MC* SE* = .07, 95% MC CI [−0.360, −0.103], and perceived expertise, *B* = −.19, MC* SE* = .07, 95% MC CI [−0.326, −0.054]. With these indirect effects taken into account, the direct effect between external (compared with internal) criticism and agreement with the message was no longer statistically significant, *B* = .01, *SE* = .11, 95% CI [−0.215, 0.225], see Figure 3.

## Discussion

Study 2 found additional evidence in favour of a norm against external criticism. Similar to Study 1, people agreed more with internal than external criticism and were more permissive and less restrictive towards internal than external critics. Additionally, perceived constructiveness of the criticism and perceived expertise of the critic mediated the effects of internal versus external criticism. Importantly and in support of a norm of preferring internal over external criticism, these findings emerged for all internal critics. Thus, perceived constructiveness and expertise were used not only to evaluate members of one’s own group who criticized internally (ethnic Dutch), but also to evaluate outgroup members (Middle Easterner) criticizing the outgroup.

## STUDY 3

The pattern of findings in Studies 1 and 2 support the norm of preferring internal over external criticism. However, some of the effects were rather weak and this might be due to a limitation in both studies. In the previous experiments, the ethnic Middle Eastern critic was described as having been born and raised in the Netherlands and it was not indicated whether his family had a refugee background. Although in Study 2 the ethnic Middle Eastern critic was perceived as having a more positive attitude towards refugees that the Dutch critic, it might be that this critic was not fully considered a member of the ‘refugee’ group. Therefore, in Study 3 we described the ethnic Middle Eastern critic as being a former refugee. We again tested the mediating mechanisms from Study 2.

## Method

### Participants

A total of 399 responses were collected in a larger Internet survey on immigration and societal attitudes.9Other topics covered in the survey included Brexit attitudes, intergroup attitudes, social tolerance beliefs, and the slippery slope fallacy. Thirty participants were excluded for not having both parents born in the Netherlands, leaving 369 participants for analysis (52.3% female, 47.7% male; age, *M* = 50.6, *SD* = 17.7, range = 18–91).

### Measures

The experimental manipulation in Study 3 was similar to those of the previous studies with one crucial change. Rather than being described as an ethnic Middle Eastern critic who was born, raised, and working in the Netherlands, this critic was described as being a former refugee with no other information about upbringing and employment status. To match this description and to remove possible confounds, the ethnic Dutch critic also was not described as having been born, raised, or working in the Netherlands. All measures were the same as in Study 2.

### Manipulation check

Critics who blamed refugees (*M* = 2.87, *SD* = 1.51) were considered to have less positive feelings towards refugees than those who blamed the Dutch (*M* = 4.85, *SD* = 1.62), *F*(1, 365) = 152.42, *p* < .001, $\eta_{p}^{2}$ = .295. Results also indicated that the Middle Eastern critic was thought to have more positive feelings towards refugees than the Dutch critic, (*M*_Middle Eastern_ = 4.02, *SD* = 1.73; *M*_Dutch_ = 3.57, *SD* = 1.93), *F*(1, 365) = 7.66, *p* = .006, $\eta_{p}^{2}$ = .021. Again, an interaction between the target of the criticism and critic identity, *F*(1, 365) = 6.47, *p* = .011, $\eta_{p}^{2}$ = .017, indicated that while both critics who blamed the Dutch were considered to have equally positive feelings towards refugees (*M*_Middle Eastern_ = 4.87, *SE* = .17; *M*_Dutch_ = 4.83, *SE* = .16), *t*(365) = 0.15, *p* = .878, *d* = .02, Dutch critics who blamed refugees were thought to have more negative feelings (*M* = 2.44, *SE* = .15) than Middle Eastern critics who blamed refugees (*M* = 3.30, *SE* = .16), *t*(365) =  −3.89, *p* < .001, *d* = −.41.

## Results

The message was again found to be generally disagreeable (*M* = 3.14, *SD* = 1.56), although participants were neutral about permitting the message (*r* = .70, *M* = 4.29, *SD* = 1.46) and were slightly in favour of restricting the message (*r* = .76, *M* = 4.71, *SD* = 1.41). In general, participants also did not consider the message to be very constructive (*M* = 3.10, *SD* = 1.60), nor that the critic had expertise (*M* = 3.43, *SD* = 1.48). Participants again highly identified as ethnic Dutch (*M* = 8.37, *SD* = 1.49). The main results were consistent even when Ethnic Dutch identification and political self‐placement were included.10Similar to the previous studies, there was weak evidence that political self‐placement interacted with the identity of the target of the criticism such that liberals preferred when the Dutch were blamed and conservatives preferred when refugees were blamed.


**Figure 4:**
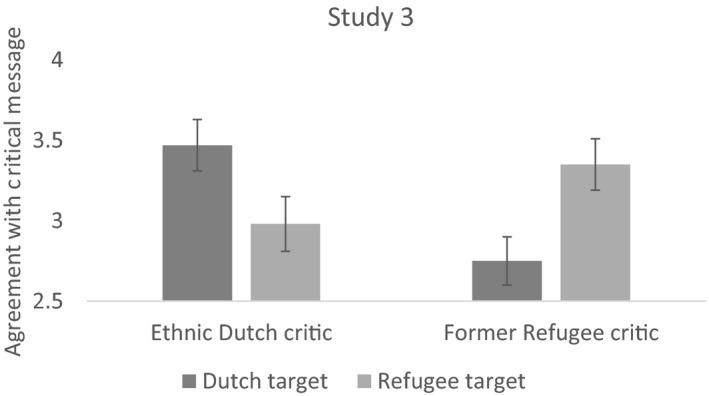
The pattern of findings for intragroup criticism (an ethnic Dutch criticizing the Dutch or a former refugee criticizing refugees) and intergroup criticism (an ethnic Dutch criticizing refugees or a former refugee criticizing the Dutch). Error bars represent standard errors (*SE*), and the outcome variable (*y*‐axis) was measured on a 1–7 scale.

ANOVAs of the critic ethnicity by target group interaction showed that internal critics were evaluated more positively than external critics (see Table 1 and Figure 4). We again created a variable that distinguished between internal and external critics, and found that participants agreed more with internal (*M* = 3.41, *SD* = 1.49) than external (*M* = 2.87, *SD* = 1.58) critics, *t*(367) = 3.33, *p* < .001, *d* = .35. They also permitted internal critics (*M* = 4.62, *SD* = 1.37) more than external critics (*M* = 3.95, *SD* = 1.49), *t*(367) = 4.45, *p* < .001, *d* = .46 and were less restrictive of internal (*M* = 4.53, *SD* = 1.39) compared with external (*M* = 4.89, *SD* = 1.42) criticism, *t*(367) = −2.45, *p* = .015, *d* = −.26. Furthermore, internal critics (*M* = 3.48, *SD* = 1.53) were judged to be more constructive than external critics (*M* = 2.71, *SD* = 1.57), *t*(367) = 4.77, *p* < .001, *d* = .50, and internal critics were considered to have more expertise (*M* = 3.72, *SD* = 1.44) than external critics (*M* = 3.14, *SD* = 1.47), *t*(367) = 3.82, *p* < .001, *d* = .40.

**Table 1:** Tests of the simple effects for the critic identity × target identity interaction in Experiment 3

Dependent variables	Actor identity	Target identity		
		Dutch people	Refugees	
Opinion agreement	Ethnic Dutch	3.47 (.16)	2.98 (.15)	*t*(365) = 2.19, *p = *.029
	Former Refugee	2.75 (.17)	3.35 (.16)	*t*(365) = −2.62, *p *= .009
		*t*(365) = 3.09, *p = *.002	*t*(365) = −1.68, *p *= .095	
Permitting the message	Ethnic Dutch	4.56 (.15)	4.16 (.14)	*t*(365) = 1.93, *p = *.055
	Former Refugee	3.70 (.16)	4.66 (.14)	*t*(365) = −4.52, *p < *.001
		*t*(365) = 3.95 *p *< .001	*t*(365) = −2.47, *p = *.014	
Restricting the message	Ethnic Dutch	4.53 (.15)	4.60 (.14)	*t*(365) = −0.33, *p = *.738
	Former Refugee	5.24 (.15)	4.54 (.14)	*t*(365) = 3.41, *p *< .001
		*t*(365) = −3.37, *p *< .001	*t*(365) = 0.30, *p *= .761	
Message constructiveness	Ethnic Dutch	3.53 (.17)	2.74 (.16)	*t*(365) = 3.48, *p < *.001
	Former Refugee	2.67 (.17)	3.44 (.16)	*t*(365) = −3.29, *p *= .001
		*t*(365) = 3.59, *p < *.001	*t*(365) = −3.16, *p *= .002	
Actor expertise	Ethnic Dutch	3.83 (.15)	2.98 (.15)	*t*(365) = 4.01, *p < *.001
	Former Refugee	3.33 (.16)	3.61 (.15)	*t*(365) = −1.32, *p = *.186
		*t*(365) = 2.28, *p *= .023	*t*(365) = −3.06, *p = *.002	

Note

The *t* tests beneath the columns compare reactions to in‐group versus outgroup critics within that column. The *t* tests to the right of the rows compare reactions to threat or no threat across the row. As the mean values represent least‐squares means (LS means), the values in parentheses represent standard errors.

### Mechanism for agreement with the critical message

Once again we tested the mechanisms of constructiveness and expertise. For brevity, we again only report the results for agreement here (see Appendix S1).

**Figure 5:**
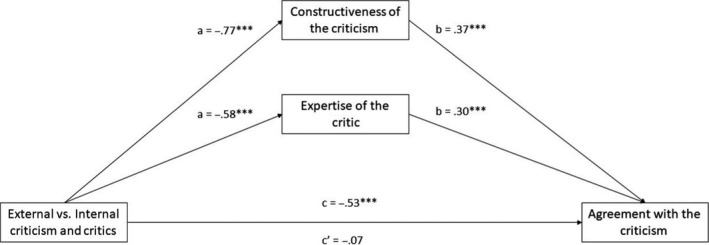
The total path of external versus internal criticism on agreement with the criticism (c path), the indirect paths leading to the two mediators (a paths), and then to the outcome variables (b paths), and the direct path from external (compared with internal) criticism on the outcome controlling for the mediators (c′ path). *p < .05, **p < .01, ***p < .001.

The total effect of external (vs. internal) criticism decreased agreement with the criticism, *B* = −.53, *SE* = .16, 95% CI [−0.847, −0.218]. As before, this effect was mediated, with external criticism considered less constructive, *B* = −.77, *SE* = .16, 95% CI [−1.091, −0.454], and coming from someone with less expertise, *B* = −.58, *SE* = .15, 95% CI [−0.877, −0.281]. In turn, constructiveness, *B* = .37, *SE* = .05, 95% CI [0.259, 0.472], and expertise, *B* = .30, *SE* = .06, 95% CI [0.189, 0.417], predicted greater agreement with the criticism (Figure 5). The direct effect was no longer significant, *B* = −.07, *SE* = .13, 95% CI [−0.336, 0.186]. Monte Carlo simulations showed significant indirect effects of constructiveness, *B* = −.28, MC* SE* = .07, 95% MC CI [−0.434, −0.147], and expertise, *B* = −.18, MC* SE* = .06, 95% MC CI [−0.304, −0.073].

## Discussion

Using a more precise in‐group versus outgroup experimental manipulation than in Studies 1 and 2, we found a similar and stronger pattern of findings in support of the norm preferring internal over external criticism. People appear to evaluate critics based on the norm that internal criticism is more acceptable than external criticism, both for their own group and for outgroups. Mediational analyses again suggest that perceptions of internal criticism as being more constructive and internal critics as having more expertise partially explain these responses.

### Sensitivity analyses

We conducted a sensitivity power analyses to determine the minimal detectable effect of the conducted studies (Erdfelder, Faul, & Buchner, 1996). We focused on the three key dependent variables of agreement, permission, and restriction of the criticism. The sensitivity for the two‐way interaction between the identity of the critic and the identity of the target was (in $\eta_{p}^{2}$) .013 in Experiment 1, .027 in Experiment 2, and .029 in Experiment 3. When looking at the comparison between internal and external critics (two groups), sensitivity was .010 in Experiment 1, .019 in Experiment 2, and .021 in Experiment 3.

The effect size found for the two‐way interactions (in $\eta_{p}^{2}$) ranged from .016 to .034 in Experiment 1, .013 to .017 in Experiment 2, and .019 to .054 in Experiment 3. These effects suggest sufficient power in Experiments 1 and 3, and weak power in Experiment 2. The effect sizes obtained for the (two groups) internal–external comparisons ranged from .017 to .033 in Experiment 1, .012 to .017 in Experiment 2, and .017 to .050 in Experiment 3, suggesting the same as above.

## GENERAL DISCUSSION

The consequences of the refugee crisis have been severe and impacted many European societies, including public debate. In three experiments, we tested whether when evaluating group criticism people are operating more based on in‐group favouritism where they are accepting of criticism from in‐group members regardless of the target or on a norm of preferring internal over external criticism regardless of the critic and target. We asked Dutch participants to react to a poster that blames the societal consequences of the refugee crisis on either the Dutch or on refugees, and being expressed by either an ethnic Dutch or Middle Eastern/refugee critic. Thus, we used a design that included not only intergroup contexts but also in‐group and outgroup contexts. This design allowed us to demonstrate that protecting the in‐group from external criticism is not a position that is only applied when one’s own group is targeted (Dutch), but is also applied when evaluating criticism targeting the outgroup. This is an important finding and indicates that it is useful for research on group criticism to take a broader perspective than the common distinction between in‐group criticism by in‐group or outgroup critics.

People generally seem to prefer internal over external criticism, regardless of their own group membership and regardless of their level of in‐group identification. They do so even when that means that their own group received blame and even when it limits their fellow in‐group members’ ability to voice an opinion critical of the reception of refugees. This general higher willingness to protect the critical speech of a person criticizing their own group has also been found among White participants in the United States and independently of individual differences in political orientation and implicit preferences for Whites (Lindner & Nosek, 2009). Furthermore, this norm seems in part to arise from expectation that internal critics are more constructive and knowledgeable than external critics. In Studies 2 and 3, perceived constructiveness and expertise were not only important for evaluating a fellow in‐group member (Dutch) criticizing the in‐group (Dutch), but also for an outgroup member (Middle Easterner/former refugee) criticizing the outgroup (refugees). Importantly, these effects emerged in experiments conducted on the politically heated topic of the refugee crisis, which has sparked intense debates within and between European countries and served as a flashpoint for political debate and national upheaval. This context raises two possibilities for the generalizability of these effects to less heated situations that should be examined empirically. From one perspective, the very heatedness of this situation might be what makes people stick to this internal over external norm to avoid making a bad situation worse. Alternatively, the maintenance of an ‘internal but not external’ norm under such heated and critical circumstances might mean that it will be even more easily maintained in calmer settings where less is at stake. This latter possibility is in line with research by Sutton Elder and Douglas (2006) into a less heated context where they found support for a norm of preferring internal over external criticism.

Although our findings indicate that people often appear to be following a norm that internal criticism is more acceptable than external criticism, social identity concerns were not entirely absent (Tajfel & Turner, 1979). In Study 1, and on support for restricting the criticism in Study 3, a preferential evaluation and treatment of fellow in‐group members appeared. People agreed more with a critic of their own group (Dutch vs. Middle Eastern) and were more willing to permit and protect his freedom of expression. This suggests that in‐group preferences can limit the extent to which people will treat internal and external critics, regardless of their own group membership, alike, and that even with a norm of internal over external criticism activated, other values, biases, and priorities affect people’s behaviour. Similarly, mixed evidence suggests that gender, for example, may affect this process, with results in one study showing that while men displayed a preference for internal over external criticism, women did not. Thus, the norm of preferring internal criticism may not be activated identically among members of different social groups.

### Limitations and future directions

There are some limitations to this research that provide directions for further exploration. First, we should note that people seemed to disagree with the message overall. This is an issue that emerges with much of the literature on group criticism, where the experimental manipulations tend to lack nuance and may not reflect responses to more balanced criticism (Adelman & Dasgupta, 2019; Hornsey *et al.*, 2002). However, much of the societal debate about the reception of refugees and immigrants tends to be characterized by a lack of nuance (Arlt & Wolling, 2018; Greussing & Boomgaarden, 2017) and our experimental manipulation was modelled on a newspaper‐style article about a realistic situation. Yet, for theoretical and applied reasons future research may want to also examine situations involving more balanced criticisms.

Second, it is possible that what we are referring to as a norm of preferring internal over external criticism may represent the meeting of two different processes: the value of protecting and favouring the in‐group and the value of protecting the weak (in this case the refugee outgroup). Research indicates that there is a norm of protecting and not criticizing weak groups, termed the ‘David and Goliath’ principle (Jeffries, Hornsey, Sutton, Douglas, & Bain, 2012). In the context of this research, that might mean that when it comes to criticizing a powerful in‐group, the ‘David and Goliath’ principle is not relevant, leading to preferential treatment of the in‐group critic. In contrast, when criticizing the weak outgroup, the principle is relevant which prevents criticism of the weaker outgroup, whereas no such effect would emerge if the outgroup was equally or more powerful. However, this explanation may conflict with the finding in the present research that the protection of weaker group does not extend to criticisms of the weaker group by members of that same group. The norm that ‘one should only criticize one’s own group’ therefore seems to provide a more parsimonious explanation of our pattern of findings. However, future research can investigate issues of power in reactions to group‐directed criticisms, especially in the context of heated political debates.

A third limitation is that while the evidence is strong for the intergroup sensitivity effect when the in‐group is targeted, the evidence was somewhat weaker when the outgroup was being targeted. When the in‐group was targeted, in‐group critics were far more acceptable than those from the outgroup. But when the outgroup was targeted, the pattern was consistent with the norm against external criticism in direction, but was weaker. This may be due to a number of causes. First, as people generally agreed that refugees were not to blame for the crisis overall (main effects), agreement with criticism of refugees may have been muted enough to similarly mute the effects. Second, perhaps rather than one process or the other being activated, it may be that both processes, in‐group protection consistent with social identity theory and the norm of preferring internal over external criticism, are simultaneously active in determining reactions to group criticism as a function of the identity of the critic. Future research can investigate this further.

Fourth, it is important to recognize that while the evidence generally indicates a norm of internal and not external criticism, social identity effects were not entirely absent. This suggests that two processes can be at play, and the importance of both might differ for example for whether people are bystanders or targets of the criticism. As bystanders, they may be concerned about conflicts that can arise from external criticism and therefore support internal but not external criticism, even if it requires limiting the ability of fellow in‐group members to engage in external criticism. As targets, social identity concerns may lead to rejecting external critics more than internal critics. Future research could try to examine when and why one or the other process is more important and how these processes might interact.

We should also note that while the manipulation checks used in Studies 2 and 3 enabled us to determine that the manipulations of criticism valence and critic identity were successfully recognized by the participants, the target group in these checks was refugees. As we did not assess how participants thought that the critic felt towards Dutch people, we do not know whether the manipulation was similarly successful for them. Further, the measure of critics’ constructiveness used in Studies 2 and 3 was a single item that asks about the perceived constructiveness of the message and not about the perceived constructive motives of the critic. Both are likely to be quite similar and future research could examine this by expanding the scale of constructiveness to include both aspects and determine whether they measure similar constructiveness judgements with similar effects.

### Conclusion

Democratic societies must engage in difficult debates, and this makes it important that people are open to controversial messages. The results of this research suggest that people use a norm that prefers internal over external criticism that can interfere with the free exchange of ideas and opinions that are key to the democratic process. While previous research indicates that group and situational biases affect what we are willing to listen to and from whom, the current research further suggests that it can be difficult to create situations in which people will be permitted to engage freely in these difficult conversations. Our research extends our understanding of the processes and expectations that are involved in restricting group criticism, and future research should further investigate these limitations and seek potential interventions to increase people’s willingness to hear and permit difficult national conversations on controversial topics.

## Author contributions

Levi Adelman, Maykel Verkuyten: Conceptualization, Data curation, Formal analysis, Funding acquisition, Investigation, Methodology, Project administration, Resources, Supervision, Writing – original draft, Writing – review & editing.

## Supporting information

Appendix S1. Manipulations, measures, correlations, and additional results.

## Data Availability

The data that support the findings of this study are openly available in OSF at https://osf.io/5zfhs/
