# Supplementary material for: Rules of engagement: Reactions to internal and external criticism in public debate
Source: Br J Soc Psychol. 2019 Nov 6;59(2):405–24. doi: 10.1111/bjso.12351 (PMC7187232; doi:10.1111/bjso.12351)
Supplement: Supplementary file 1 — Appendix S1. Manipulations, measures, correlations, and additional results. [file BJSO-59-405-s001.docx]

Rules of Engagement:

Reactions to Internal and External Criticism in Public Debate

**Appendix**

**Table of contents**

1. **Study manipulations and measures**
2. **Additional results from Study 2**
3. **Additional results from Study 3**
4. **Correlations**
5. **Testing the effect of Gender**

**Manipulations and measures**

**Study 1**

*Manipulations*

Gouda - Johan Kok, aged 43, stuck a poster on the window of the community center with "THE DUTCH ARE THE PROBLEM" in large block letters. Kok, born and raised in the Netherlands and working for 20 years at the same technical company explained his action by saying "people must realize who is responsible here". His local residents disagreed and found the poster 'unnecessarily offensive and very hurtful' and asked him to remove the poster.

Gouda - Johan Kok, aged 43, stuck a poster on the window of the community center with large letters in it "REFUGEES ARE THE PROBLEM". Kok, born and raised in the Netherlands and working for 20 years at the same technical company explained his action by saying "people must realize who is responsible here". His local residents disagreed and found the poster 'unnecessarily offensive and very hurtful' and asked him to remove the poster.

Gouda - Yusuf Hamdi, aged 43, has posted a poster on the window of the community center with the letters "THE DUTCH ARE THE PROBLEM" in large block letters. Hamdi, born and raised in the Netherlands and working for 20 years at the same technical company explained his action by saying "people must realize who is responsible here". His local residents disagreed and found the poster 'unnecessarily offensive and very hurtful' and asked him to remove the poster.

Gouda - Yusuf Hamdi, aged 43, stuck a poster on the window of the community center with large letters in it "REFUGEES ARE THE PROBLEM". Hamdi, born and raised in the Netherlands and working for 20 years at the same technical company explained his action by saying "people must realize who is responsible here". His local residents disagreed and found the poster 'unnecessarily offensive and very hurtful' and asked him to remove the poster.

*Measures*

Opinion Agreement:

I agree with his opinion.

Permitting the message:

It must be allowed for him to give his opinion in this way.

The local residents must accept his action.

Restricting the message:

He should remove the poster to prevent neighbors from feeling offended and hurt.

He should remove the poster to prevent tensions and conflicts in the neighborhood

Ethnic Dutch Identification:

How strongly do you feel Dutch?

**Study 2**

*Manipulations*

Gouda - Johan Kok, aged 43, stuck a poster on the window of the community center with "THE DUTCH ARE THE PROBLEM" in large block letters. Kok, born and raised in the Netherlands and working for 20 years at the same technical company explained his action by saying "people must realize who is responsible here". His local residents disagreed and found the poster 'unnecessarily offensive and very hurtful' and asked him to remove the poster.

Gouda - Johan Kok, aged 43, stuck a poster on the window of the community center with large letters in it "REFUGEES ARE THE PROBLEM". Kok, born and raised in the Netherlands and working for 20 years at the same technical company explained his action by saying "people must realize who is responsible here". His local residents disagreed and found the poster 'unnecessarily offensive and very hurtful' and asked him to remove the poster.

Gouda - Yusuf Hamdi, aged 43, has posted a poster on the window of the community center with the letters "THE DUTCH ARE THE PROBLEM" in large block letters. Hamdi, born and raised in the Netherlands and working for 20 years at the same technical company explained his action by saying "people must realize who is responsible here". His local residents disagreed and found the poster 'unnecessarily offensive and very hurtful' and asked him to remove the poster.

Gouda - Yusuf Hamdi, aged 43, stuck a poster on the window of the community center with large letters in it "REFUGEES ARE THE PROBLEM". Hamdi, born and raised in the Netherlands and working for 20 years at the same technical company explained his action by saying "people must realize who is responsible here". His local residents disagreed and found the poster 'unnecessarily offensive and very hurtful' and asked him to remove the poster.

*Measures*

Manipulation check:

What do you think are [critic]’s feelings towards refugees?

Opinion Agreement:

I agree with his opinion.

Permitting the message:

It must be allowed for him to give his opinion in this way.

The local residents must accept his action.

Restricting the message:

He should remove the poster to prevent neighbors from feeling offended and hurt.

He should remove the poster to prevent tensions and conflicts in the neighborhood

Ethnic Dutch Identification:

How strongly do you feel Dutch?

Message constructiveness:

To what extent do you think that [critic]'s action makes a positive contribution to the debate on refugees?

Critic expertise:

To what extent do you think [critic] is well informed about the refugee issue?

**Study 3**

*Manipulations*

Gouda - Johan Kok, aged 43, stuck a poster on the window of the community center with "THE DUTCH ARE THE PROBLEM" in large block letters. Kok explained his action by saying "people must realize who is responsible here". His local residents disagreed and found the poster 'unnecessarily offensive and very hurtful' and asked him to remove the poster.

Gouda - Johan Kok, aged 43, stuck a poster on the window of the community center with large letters in it "REFUGEES ARE THE PROBLEM". Kok explained his action by saying "people must realize who is responsible here". His local residents disagreed and found the poster 'unnecessarily offensive and very hurtful' and asked him to remove the poster.

Gouda - Yusuf Hamdi, a former refugee, aged 43, stuck a poster on the window of the community center with the letters "THE DUTCH ARE THE PROBLEM" in large block letters. Hamdi explained his action by saying "people must realize who is responsible here". His local residents disagreed and found the poster 'unnecessarily offensive and very hurtful' and asked him to remove the poster.

Gouda - Yusuf Hamdi, a former refugee, aged 43, stuck a poster on the window of the community center with in large block letters "REFUGEES ARE THE PROBLEM". Hamdi explained his action by saying "people must realize who is responsible here". His local residents disagreed and found the poster 'unnecessarily offensive and very hurtful' and asked him to remove the poster.

*Measures*

Manipulation check:

What do you think are [critic]’s feelings towards refugees?

Opinion Agreement:

I agree with his opinion.

Permitting the message:

It must be allowed for him to give his opinion in this way.

The local residents must accept his action.

Restricting the message:

He should remove the poster to prevent neighbors from feeling offended and hurt.

He should remove the poster to prevent tensions and conflicts in the neighborhood

Ethnic Dutch Identification:

How strongly do you feel Dutch?

Message constructiveness:

To what extent do you think that [critic]'s action makes a positive contribution to the debate on refugees?

Critic expertise:

To what extent do you think [critic] is well informed about the refugee issue?

**Additional details from Experiment 2: The full description of the two-way interaction**

First, we tested whether we would replicate the findings of Experiment 1, and once again we found significant interactions between the group identity of the person who put up the poster and the identity of the group targeted by the message on agreement with the message, *F*(1, 400) = 6.92, *p* = .009, ηp^2^ = .017, permitting the message, *F*(1, 400) = 6.76, *p* = .010, ηp^2^ = .017, and restricting the message, *F*(1, 400) = 5.13, *p* = .024, ηp^2^ = .013. As can be seen in Table A1, the results are similar to those of Experiment 1. The non-ethnic Dutch actor tended to be better received when blaming refugees rather than the Dutch, while the Dutch actor tended to be better received when blaming the Dutch rather than refugees.

| **Table A1** |  |  |  |  |
| --- | --- | --- | --- | --- |
| *Tests of the simple effects for the Critic identity x Target identity interaction in Experiment 2.* | | | | |
|  |  | **Target Identity** | |  |
| **Dependent Variable** | **Actor Identity** | **Dutch People** | **Refugees** |  |
| Opinion Agreement | Ethnic Dutch | 3.52 (0.16) | 3.15 (0.16) | *t*(400)= 1.65, *p=*.101 |
|  | Non-Ethnic Dutch | 2.91 (0.16) | 3.36 (0.15) | *t*(400)=-2.08, *p*=.038 |
|  |  | *t*(400)=2.70, *p=*.007 | *t*(400)=-.97, *p*=.331 |  |
| Permitting the Message | Ethnic Dutch | 4.79 (0.14) | 4.37 (0.14) | *t*(400)=2.16, *p=*.031 |
|  | Non-Ethnic Dutch | 4.46 (0.14) | 4.75 (0.13) | *t*(400)=-1.50, *p=*.133 |
|  |  | *t*(400)=1.67, *p*=.095 | *t*(400)=-2.02, *p=*.045 |  |
| Restricting the Message | Ethnic Dutch | 4.38 (0.14) | 4.63 (0.14) | *t*(400)=-1.25, *p=*.212 |
|  | Non-Ethnic Dutch | 4.81 (0.14) | 4.43 (0.13) | *t*(400)=1.97, *p*=.050 |
|  |  | *t*(400)=-2.13, *p*=.034 | *t*(400)=1.04, *p*=.299 |  |
| *Note.* The *t*-tests beneath the columns compare reactions to ingroup vs. outgroup critics within that column. The *t*-tests to the right of the rows compare reactions to threat or no threat across the row. As the mean values represent least-squares means (LS means), the values in parentheses represent Standard Errors. | | | | |

**Effects on mechanism variables.** A two way interaction between actor identity and target identity, *F*(1, 400) = 14.04, *p* < .001, ηp^2^ = .034, showed that when the message came from an ethnic Dutch actor, it was perceived to be more constructive when it targeted the Dutch (*M* = 3.44, *SE* = 0.16) rather than refugees (*M* = 2.82, *SE* = 0.16), *t*(400) = 2.73, *p* = .007, *d* = 0.27. In contrast, when the message came from a non-ethnic Dutch actor, it was perceived as more constructive when it targeted refugees (*M* = 3.24, *SE* = 0.15) rather than the Dutch (*M* = 2.68, *SE* = 0.16), *t*(400) = 2.57, *p* = .011, *d* = 0.26.

Participants also differed in their perception of how informed the actor was based on the actor’s identity and target. Once again, a significant interaction, *F*(1, 400) = 8.29, *p* = .004, ηp^2^ = .020, showed that ethnic Dutch actors were judged as better informed when they blamed Dutch people (*M* = 3.78, *SE* = 0.15) rather than refugees (*M* = 3.17, *SE* = 0.14), *t*(400) = 2.98, *p* = .003, *d* = 0.30, although non-ethnic Dutch actors did not differ in judgments of how informed they were (*M*_DutchTarget_ = 3.42, *SE* = 0.14; *M*_RefugeeTarget_ = 3.62, *SE* = 0.13), *t*(400) = -1.05, *p* = .294, *d* = -0.11.

**Additional mediation results**

**Permitting the critical message**

The total effect of external (compared to internal) criticism on support for permitting the message, *B* = -.36, *SE* = .14, 95% *CI* [-.625, -.087], was also mediated with external (compared to internal) criticism leading to decreased constructiveness, *B* = -.58, *SE* = .16, 95% *CI* [-.884, -.269], and perceived expertise, *B* = -.40, *SE* = .15, 95% *CI* [-.678, -.125]. Both constructiveness, *B* = .11, *SE* = .05, 95% *CI* [.016, .209], and expertise, *B* = .32, *SE* = .05, 95% *CI* [.217, .431], then predicted more support for permitting the message. Monte Carlo simulations confirmed significant indirect effects of constructiveness, *B* = -.07, *MC SE* = .03, 95% *MC CI* [-.143, -.008], and expertise, *B* = -.13, *MC SE* = .05, 95% *MC CI* [-.238, -.038]. In accounting for the indirect effects, the direct effect between external (compared to internal) criticism and support for permitting the message was no longer significant, *B* = -.16, *SE* = .13, 95% *CI* [-.410, .088].

**Restricting the critical message**

The total effect on restricting the message, *B* = .31, *SE* = .14, 95% *CI* [.039, .588], was mediated with external (compared to internal) criticism decreasing constructiveness, *B* = -.58, *SE* = .16, 95% *CI* [-.884, -.269], and expertise, *B* = -.40, *SE* = .15, 95% *CI* [-.678, -.125]. However, only the perceived expertise of the critic, *B* = -.26, *SE* = .06, 95% *CI* [-.369, -.141], but not constructiveness, *B* = -.08, *SE* = .05, 95% *CI* [-.182, .024], predicted less support for restricting the message. Accounting for the indirect effects, the direct effect was no longer significant, *B* = .17, *SE* = .14, 95% *CI* [-.100, .431], with an overall indirect effect of expertise, *B* = .10, *MC SE* = .04, 95% *MC CI* [.028, .197], but not of constructiveness, *B* = .05, *MC SE* = .03, 95% *MC CI* [-.012, .121].


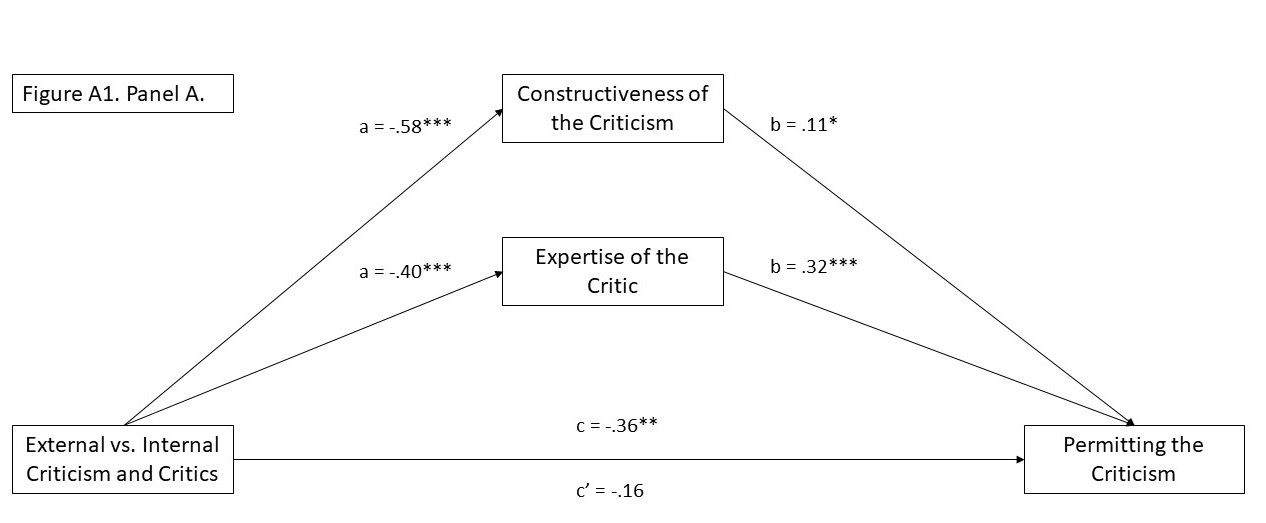


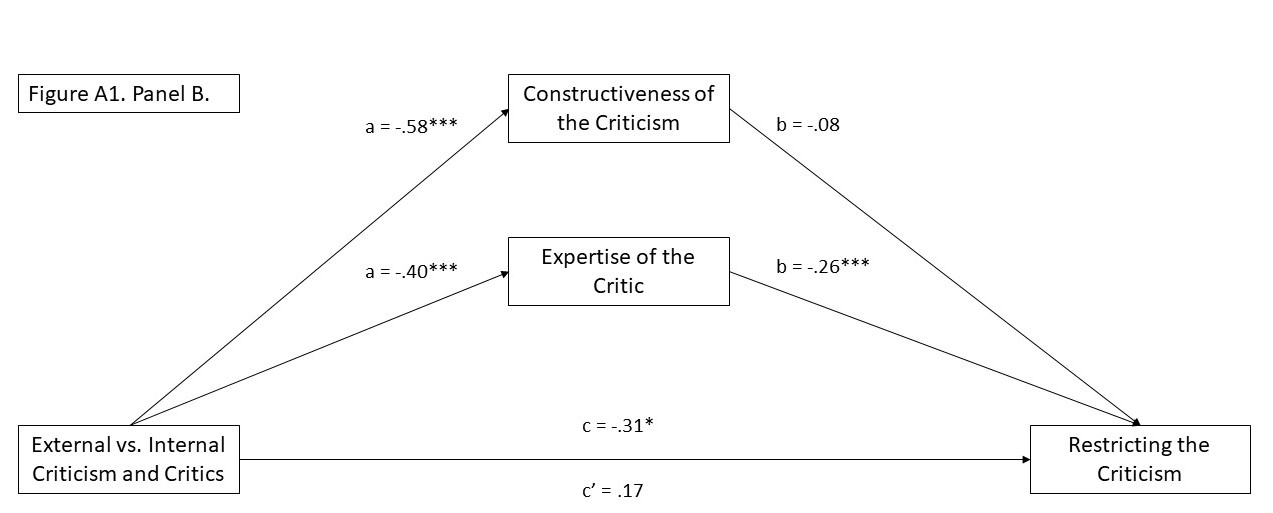


Figure A1, panels A-B. The total path of external vs. internal criticism on permitting, and restricting the criticism (c path), the indirect paths leading to the two mediators (a paths), and then to the outcome variables (b paths), and the direct path from external (compared to internal) criticism on the outcome controlling for the mediators (c’ path).

**Additional results from Experiment 3: The description of the full interaction**

To test whether we would find a similar pattern of effects when the actor was explicitly described as a former refugee, we conducted the same ANOVAs as in Experiment 2. Once again significant interactions between actor identity and target identity emerged for agreement with the message, *F*(1, 365) = 11.58, *p* < .001, ηp^2^ = .031, permitting the message, *F*(1, 365) = 20.92, *p* < .001, ηp^2^ = .054, restricting the message, *F*(1, 365) = 7.12, *p* = .008, ηp^2^ = .019, perceived constructiveness, *F*(1, 365) = 22.85, *p* < .001, ηp^2^ = .059, perceived expertise, *F*(1, 365) = 14.10, *p* < .001, ηp^2^ = .037, and perceived attitudes toward refugees, *F*(1, 365) = 6.47, *p* = .011, ηp^2^ = .017.
 As Table A2 shows, Experiment 3 replicates the findings of Experiments 1 and 2, but more strongly. From one perspective, looking at the first column, when the Dutch are blamed there is a consistently more positive response when the actor is ethnic Dutch and not a former refugee. In contrast, looking at the second column, the former refugee is preferred as the messenger when blaming refugees, evaporating and reversing the ingroup benefit granted to the ethnic Dutch actor. From another perspective, looking down the rows, when the actor is Dutch, Dutch participants prefer that they blame Dutch rather than refugees. But when the actor is a former refugee, they are preferred to be blaming refugees and not the Dutch.

| **Table A2** |  |  |  |  |
| --- | --- | --- | --- | --- |
| *Tests of the simple effects for the Critic identity x Target identity interaction in Experiment 3.* | | | | |
|  |  | **Target Identity** | |  |
| **Dependent Variable** | **Actor Identity** | **Dutch People** | **Refugees** |  |
| Opinion Agreement | Ethnic Dutch | 3.47 (0.16) | 2.98 (0.15) | *t*(365)= 2.19, *p=*.029 |
|  | Former Refugee | 2.75 (0.17) | 3.35 (0.16) | *t*(365)=-2.62, *p*=.009 |
|  |  | *t*(365)=3.09, *p=*.002 | *t*(365)=-1.68, *p*=.095 |  |
| Permitting the Message | Ethnic Dutch | 4.56 (0.15) | 4.16 (0.14) | *t*(365)=1.93, *p=*.055 |
|  | Former Refugee | 3.70 (0.16) | 4.66 (0.14) | *t*(365)=-4.52, *p<*.001 |
|  |  | *t*(365)=3.95 *p*<.001 | *t*(365)=-2.47, *p=*.014 |  |
| Restricting the Message | Ethnic Dutch | 4.53 (0.15) | 4.60 (0.14) | *t*(365)=-0.33, *p=*.738 |
|  | Former Refugee | 5.24 (0.15) | 4.54 (0.14) | *t*(365)=3.41, *p*<.001 |
|  |  | *t*(365)=-3.37, *p*<.001 | *t*(365)=0.30, *p*=.761 |  |
| Message Constructiveness | Ethnic Dutch | 3.53 (0.17) | 2.74 (0.16) | *t*(365)=3.48, *p<*.001 |
|  | Former Refugee | 2.67 (0.17) | 3.44 (0.16) | *t*(365)=-3.29, *p*=.001 |
|  |  | *t*(365)=3.59, *p<*.001 | *t*(365)=-3.16, *p*=.002 |  |
| Actor Expertise | Ethnic Dutch | 3.83 (0.15) | 2.98 (0.15) | *t*(365)=4.01, *p<*.001 |
|  | Former Refugee | 3.33 (0.16) | 3.61 (0.15) | *t*(365)=-1.32, *p=*.186 |
|  |  | *t*(365)=2.28, *p*=.023 | *t*(365)=-3.06, *p=*.002 |  |
| Actor Attitudes | Ethnic Dutch | 4.83 (0.16) | 2.44 (0.15) | *t*(365)=10.64, *p<*.001 |
|  | Former Refugee | 4.87 (0.17) | 3.30 (0.16) | *t*(365)=6.86, *p*<.001 |
|  |  | *t*(365)=-0.15, *p*=.878 | *t*(365)=3.89, *p*<.001 |  |
| *Note.* The *t*-tests beneath the columns compare reactions to ingroup vs. outgroup critics within that column. The *t*-tests to the right of the rows compare reactions to threat or no threat across the row. As the mean values represent least-squares means (LS means), the values in parentheses represent Standard Errors. | | | | |

**Tests of the Mechanism**

Tests of mechanism were performed using the Process macro for SAS (Hayes, 2013).

**Permitting the critical message**

Once again, the significant total effect on permitting the criticism, *B* = -.66, *SE* = .15, 95% *CI* [-.954, -.369], was mediated through decreased constructiveness, *B* = -.77, *SE* = .16, 95% *CI* [-1.091, -.454], and perceived expertise, *B* = -.58, *SE* = .15, 95% *CI* [-.877, -.281], and then from constructiveness, *B* = .18, *SE* = .06, 95% *CI* [.069, .296], and expertise, *B* = .24, *SE* = .06, 95% *CI* [.114, .356], to permitting the criticism. The direct effect was reduced but still significant, *B* = -.38, *SE* = .14, 95% *CI* [-.662, -.107]. Monte Carlo simulations then estimated the indirect effects of constructiveness, *B* = -.14, *MC SE* = .05, 95% *MC CI* [-.258, -.047], and expertise, *B* = -.14, *MC SE* = .05, 95% *MC CI* [-.248, -.050].

**Restricting the critical message**

The total effect on restricting the criticism, *B* = .36, *SE* = .15, 95% *CI* [.071, .645], was mediated through constructiveness, *B* = -.77, *SE* = .16, 95% *CI* [-1.091, -.454], and perceived expertise, *B* = -.58, *SE* = .15, 95% *CI* [-.877, -.281]. However, only constructiveness, *B* = -.18, *SE* = .06, 95% *CI* [-.295, -.060], but not expertise, *B* = -.07, *SE* = .06, 95% *CI* [-.191, .060], predicted less support for restricting the message. The remaining direct effect was no longer significant, *B* = .18, *SE* = .15, 95% *CI* [-.105, .471], with an overall indirect effect of constructiveness, *B* = .14, *MC SE* = .05, 95% *MC CI* [.042, .256], but not expertise, *B* = .04, *MC SE* = .04, 95% *MC CI* [-.035, .122].


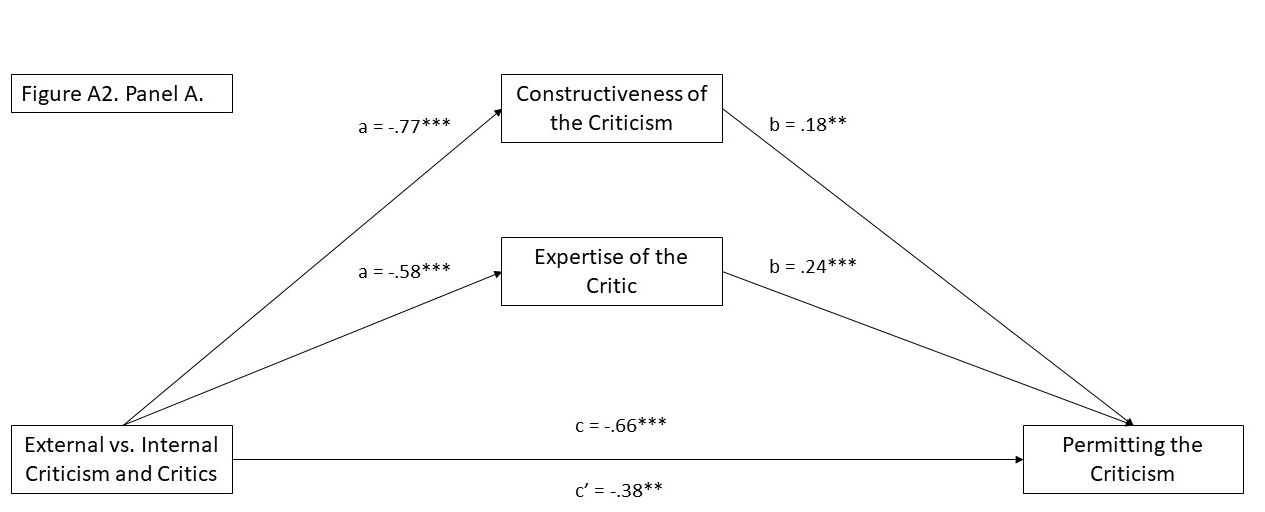


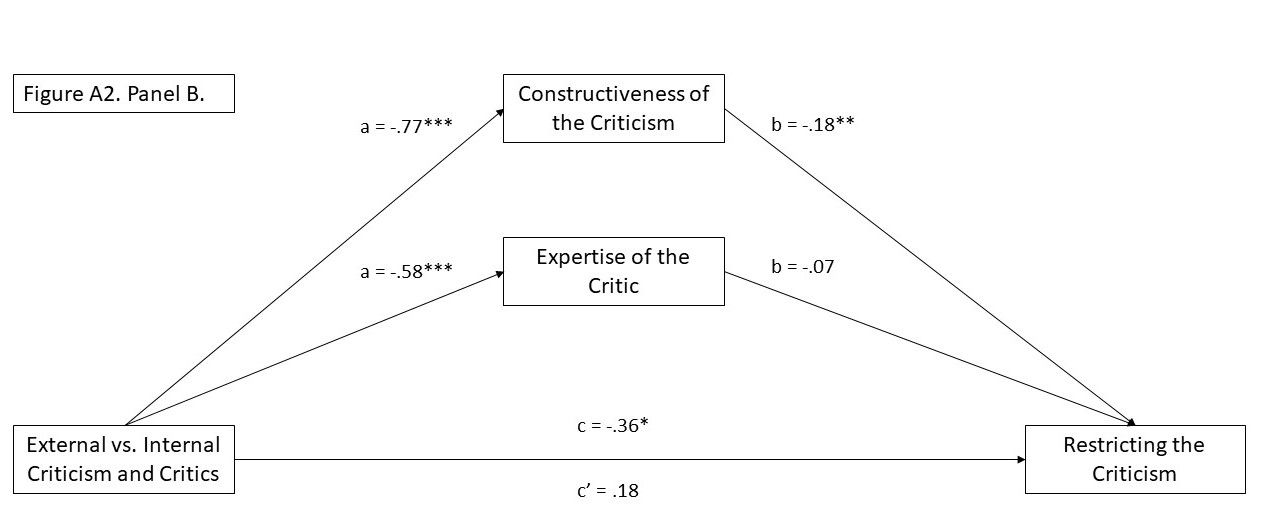


Figure 2, panels A-B. The total path of external vs. internal criticism on permitting and restricting the criticism (c path), the indirect paths leading to the two mediators (a paths), and then to the outcome variables (b paths), and the direct path from external (compared to internal) criticism on the outcome controlling for the mediators (c’ path).

**Correlations**

**Study 1**

Agreement with the actor’s opinion was positively correlated to permitting the message, *r* = .51, *p* < .001, and negatively associated with restricting the message by taking down the poster, *r* = -.59, *p* < .001. Measures of permitting and restricting the message were also significantly negatively correlated, *r* = -.41, *p* < .001.

**Study 2**

| Table A2 |  |  | |  |  |  |  |  |  | |
| --- | --- | --- | --- | --- | --- | --- | --- | --- | --- | --- |
|  |  | | *Correlations between mediating and outcome variables in Experiment 2.* | | | | | | | |
|  |  | Message Agreement | | Permitting Message | Restricting Message | Constructive Message | Actor Expertise | Ethnic Dutch Identification | |  |
| Message Agreement |  | -- | |  |  |  |  |  |  | |
| Permitting Message |  | .39*** | | -- |  |  |  |  |  | |
| Restricting Message |  | -.34*** | | -.43*** | -- |  |  |  |  | |
| Constructive Message |  | .64*** | | .34*** | -.25*** | -- |  |  |  | |
| Actor Expertise |  | .65*** | | .42*** | -.32*** | .56*** | -- |  |  | |
| Ethnic Dutch Identification |  | .04 | | .04 | .11* | .07 | -.00 |  | -- | |

**Study 3**

| Table A3 |  |  |  |  |  |  |  |  |
| --- | --- | --- | --- | --- | --- | --- | --- | --- |
|  | *Correlations between mediating and outcome variables in Experiment 3.* | | | | | | |  |
|  |  | Message Agreement | Permitting Message | Restricting Message | Constructive Message | Actor Expertise | Ethnic Dutch Identification |  |
| Message Agreement |  | -- |  |  |  |  |  |  |
| Permitting Message |  | 0.38*** | -- |  |  |  |  |  |
| Restricting Message |  | -.34*** | -.44*** | -- |  |  |  |  |
| Constructive Message |  | .57*** | .39*** | -.26*** | -- |  |  |  |
| Actor Expertise |  | .54*** | .40*** | -.22*** | .66*** | -- |  |  |
| Ethnic Dutch Identification |  | -.09 | -.07 | .06 | -.02 | -.08 | -- |  |
|  |  |  |  |  |  |  |  |  |

**Testing the Effect of Gender**

One assumption of a principle of internal and not external criticism is that it is also shared across some general demographic social groups. Gender is of particular interest, as men and women may differ in terms of their roles in enforcing group norms and boundaries, which might in turn make them differently open to internal and external criticism. Therefore, we conducted analysis across the three studies to identify the role of participant gender in reactions to group criticism.

In Experiments 1 (n = 808) and 3 (n = 369), there were no interactions between whether the criticism was internal or external and gender, all *Fs* < 1.35, all *p*s > .246. In Experiment 2 (n = 404), there was one significant interaction on acceptance of the critical opinion, *F*(3, 400) = 4.88, *p* = .028, such that men were more accepting of an internal criticism than an external criticism (LSM_Internal_ = 4.89, LSM_External_ = 4.23), whereas women did not differ (LSM_Internal_ = 4.64, LSM_External_ = 4.59).

These effects support the proposal that is a general principle that appears to apply across gender groups. At the same time, the results from Experiment 2 suggest that gender may play a weak role, and future research should ensure to test effects of gender to better understand whether gender groups differ in their policing of this principle.
